# Effects of *Cuminum cyminum L.* essential oil and its nanoemulsion on oxidative stability and microbial growth in mayonnaise during storage

**DOI:** 10.1002/fsn3.3457

**Published:** 2023-05-22

**Authors:** Asma Moradi, Nafiseh Davati, Aryou Emamifar

**Affiliations:** ^1^ Department of Food Science and Technology, College of Food Industry Bu‐Ali Sina University Hamedan Iran

**Keywords:** *Cuminum cyminum* L., essential oil, mayonnaise, nanoemulsion

## Abstract

The present study aimed to investigate the effects of *Cuminum cyminum L.* essential oil (CEO) and its nanoemulsion (CEON) on oxidative stability and microbial growth of mayonnaise during storage. The GC analysis indicated that Cuminaldehyde (27.99%), o‐Cymene (17.31%), γ‐Terpinen (16.67%), and β‐Pinene (9.35%) were the major components of CEO, respectively. The assessments of minimum inhibitory concentration (MIC) and minimum bactericidal concentration (MBC) showed that *Escherichia coli* ATCC 25922 (MBC_CEO_ = 12 and MBC_CEON_ = 6 mg/mL) was the most resistant bacteria, and in contrast, *Staphylococcus aureus* ATCC 29213 (MBC_CEO_ = 6 and MBC_CEON_ = 3 mg/mL) was the most sensitive bacteria. In the radical‐scavenging assay, CEON (IC^50^ = 5 ± 0.07 μg/mL) exhibited a higher antioxidant activity than CEO (IC^50^ = 10 ± 0.13 μg/mL). The results showed that applying the MBC of CEO and CEON in mayonnaise led to a significant decrease (*p* < .05) in acidity, peroxide value, number of acid‐resistant bacteria and fungi, and total microbial count compared with the control sample. In conclusion, this study demonstrated that using CEON resulted in oxidative stability, microbial growth control, and desirable sensorial attributes in mayonnaise compared with CEO and control samples.

## Graphical abstract

Nowadays, there is a strong tendency to use natural preservatives such as essential oils in food products. *Cuminum cyminum* L. essential oil nanoemulsion (CEON) has considerable antioxidant and antimicrobial activities. CEON can more effectively result in the oxidative stability of mayonnaise compared with pure essential oil. CEON can more effectively control the microbial growth of mayonnaise compared with pure essential oil.

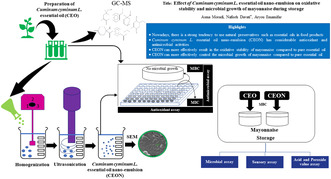

## 1 INTRODUCTION

Mayonnaise, one of the most popular sauces, is a mixture of water, egg yolk, vinegar, vegetable oil, sugar, and spices. This product is an oil‐in‐water emulsion, with a dispersed *oil* phase and a continuous *water* phase, despite 65%–85% fat content (Alizadeh et al., 2019; Depree & Savage, 2001). *Mayonnaise*, as a high‐fat food, is susceptible to chemical spoilage due to *oxidation* of the unsaturated and polyunsaturated fatty acids, since a large surface of the oil is exposed to the aqueous phase and air bubbles (Depree & Savage, 2001). Lipid oxidation of edible oils leads to the degradation of the unsaturated fatty acid and promotes off‐flavor development in *mayonnaise*. *Therefore, this* oxidation process changes aroma, texture, and color, and leads to the destruction of beneficial polyunsaturated fatty acids in the product. Additionally, due to the formation of harmful compounds like peroxidase, lipid oxidation is dangerous for humans (Kwon et al., 2015). Adding antioxidants to the emulsions can be one strategy to delay or limit lipid oxidation. Antioxidants can decrease oxidative damage through the deactivation of free radicals, prooxidants suppression, inhibition of the activity of enzymes producer free radicals, the boosting of the function of antioxidant enzymes, and oxidation control (Kishk & Elsheshetawy, 2013; Lu et al., 2010; Sørensen et al., 2017). In recent decades, synthetic antioxidants including butylated hydroxytoluene (BHT) and butylated hydroxyanisole (BHA) have been used to retard lipid oxidation due to their high effectiveness, affordable production, and desirable oxidative stability in comparison with natural antioxidants (Li et al., 2014). In contrast, synthetic antioxidants have disadvantages including low water solubility, safety concerns, and health issues such as *gastrointestinal* tract problems, fatty liver, skin allergies, storage in adipose tissue, and carcinogenesis (Gülçin, 2002; Lourenco et al., 2019; Valenzuela & Nieto, 1996). Recently, due to the side effects of synthetic antioxidants, there has been a high tendency to use natural antioxidants, as a suitable alternative, especially those with herb and spice origins (Alizadeh et al., 2019). *Cuminum cyminum L*. is a species of the *Apiaceae* family and indigenous to Southwest Asia and the Eastern Mediterranean countries. In many countries, cumin is widely used as an aromatic plant and spice for flavoring foods (Mandal & DebMandal, 2016). Scientific reports have shown that *Cuminum cyminum L*. and its essential oil as a natural food preservative possess antioxidant, antimicrobial, antifungal, and therapeutic properties (De et al., 2003; Mandal & DebMandal, 2016). The antioxidant activity of *Cuminum cyminum L*. essential oil (CEO) can be related to the existence of phenolic and polyphenolic compounds.

Nanoemulsions as kinetically stable colloidal systems with droplet size between 20 nm and 200 nm (Lago et al., 2019; Li & Chiang, 2012) possess functional properties and resistance to gravitational separation, coalescence, and aggregation in comparison with conventional emulsions (McClements, 2011). Essential oils nanoemulsions can be used more effectively in the food industry due to the larger contact area between bioactive compounds and food matrix than pure essential oils; consequently, their antioxidant and antimicrobial activities increase (McClements, 2011; Otoni et al., 2014). The first objective of the present study was to determine the antioxidant and antimicrobial activities of the CEO and *Cuminum cyminum L*. essential oil nanoemulsions (CEON) against food‐borne microorganisms. The second objective was to evaluate the microbial growth and oxidative stability of mayonnaise containing CEO and CEON compared with control during storage.

## 2 MATERIALS AND METHODS

### 2.1 Preparation of *Cuminum cyminum L.* essential oil

*Cuminum cyminum L*. essential oil from the Northeast region of Iran was supplied by Johare‐Taem company and stored in a dry, dark, and cool place (Mashhad, Iran).

### 2.2 Gas chromatography–mass spectrometry analysis

The chemical composition analysis of CEO was performed by gas chromatography–mass spectrometry (GC–MS) model Scion‐456‐SQ‐Netherlands (Scion, UK) using HP‐5MS capillary column, CP Sil 5 (25 m, 0.25 mm, film thickness 0.25 μm). The GC was performed at the injector temperature of 250°C, split 100 with the following conditions: helium gas as the carrier gas with a flow rate of 1 mL/min; at first, the column temperature was held at 45°C for 2 min then increased to 220°C at rate 3°C/min (hold for 5 min) and lastly to 270°C at rate 15°C/min (hold for 5 min); volume injected, 1 μL of the oil; and split ratio, 1:100. The MS operating parameters were as follows: electron energy 70 eV; source temperature 230°C; transfer line temperature 230°C; mass up to 650 resolution 0.7. The components of the CEO were identified using the retention time data from the National Institute of Standards and Technology (NIST) data collection (Kabouche et al., 2009; Sharifi et al., 2021).

### 2.3 Preparation of CEO and CEON

*Cuminum cyminum L*. essential oil nanoemulsions was prepared using the methods described by Gahruie et al. (2017) and Chu et al. (2020) at ambient temperature (approximately 22°C). CEO (disperse phase) and deionized water (continuous phase) were used to make CEON. First, tween 80 (7.5% v/v) was stirred in deionized water at room temperature (800 rpm, 30 min), and then CEO (7.5% v/v) was added. The obtained mixture was homogenized at 12,000 rpm for 4 min using a laboratory stirrer (OS20‐Pro, Dragon, China). Then, the coarse emulsion was exposed to ultrasonic for 10 min using a homogenizer in ultrasonic (probe) technology (BANDELIN SONOPULS HD 3100, BANDELIN, Germany) with a 30% power output of 225 W and 25 kHz.

### 2.4 Particle size measurement

The average droplet size (z‐average) of nanoemulsions and polydispersity index (PDI) were determined using a size analyzer model Nano‐ZS90 (Malvern, UK) at 25°C. First, CEON was diluted with deionized water to 10:1000 to avoid multiple scattering problems. The span of emulsion droplet sizes was measured using the following Equation (1).

(1) $$\text{Span}=\left(D90-D10\right)/D50$$

where D90, D50, and D10 are particle sizes of CEON corresponding to 90, 50, and 10% intensity on a relative cumulative particle size distribution curve.

### 2.5 Scanning electron microscope of CEON

The morphology of CEON was characterized using a scanning electron microscope (SEM) model Quanta 450 FEG (FEI, USA). CEON were lyophilized, then sputtered with a thin layer of gold. SEM images were taken at an operating voltage of 30.0 kV.

### 2.6 Antioxidant activity assay

The efficacy of the CEO and CEON to scavenge 2,2′‐diphenyl‐1‐picrylhydrazyl (DPPH) radicals was determined (Cuendet et al., 1997; Kirby & Schmidt, 1997). First, 25 μL of different dilutions (0.01%, 0.1%, and 1%) of each of CEO and CEON was mixed with 2.5 mL of 0.004% methanol solution of DPPH and subsequently, incubated for 30 min at ambient temperature (approximately 22°C). The absorbance of samples was measured against the control using spectrophotometry at 517 nm. The inhibition percentage was determined using Equation (2).

(2) $$\text{Inhibition percent}=\frac{\mathrm{AC}-\mathrm{AS}}{\mathrm{AC}}\times 100$$

AC = absorbance of the control (containing all reagents except the test compound); AS = absorbance of the sample.

The IC_50_ (μg/mL) is the concentration of antioxidant required for 50% DPPH free radical scavenging.

### 2.7 Antibacterial activity assay of CEO and CEON

The antibacterial activity of CEON and CEO was evaluated against *Escherichia coli* ATCC 25922, *Pseudomonas aeruginosa* ATCC 9027, *Staphylococcus aureus* ATCC 29213, and *Bacillus cereus* ATCC 11778 by broth microdilution assay. The bacterial strains were purchased from the Iranian Research Organization for Science and Technology (IROST, Iran). All bacteria were grown in Mueller Hinton broth (MHB) (Merck, Darmstadt, Germany), and incubated at 37°C. The antimicrobial assays, including minimum inhibitory concentration (MIC) and minimum bactericidal concentration (MBC), were performed according to Ferraro (2000), Herreros et al. (2005), Mayrhofer et al. (2008), and Chen et al. (2019). First, the CEON and CEO were dissolved in DMSO as treatments, and then serial dilution (0.37–24 mg/mL) was carried out in a 96‐well microplate. Each well of this plate containing 150 μL of the MHB and 150 μL of each serially diluted treatment was inoculated at 1% (v/v) with overnight cultures of test bacteria. Then, the microplates with the final concentration of bacteria of approximately 10^6^ CFU/mL were incubated at 37°C for 24 h. MIC is defined as the lowest concentration of CEON and CEO that inhibited any visible growth. The microwell that presented no visible microbial growth was cultured on Mueller Hinton agar (MHA) (Merck, Darmstadt, Germany). MBC was defined as the lowest concentration of CEON and CEO suppressing the colony‐forming ability after 24 h of incubation at 37°C.

### 2.8 Time‐kill kinetic analysis of CEO and CEON

The inhibitory effects of CEO and CEON on the growth curve of *E. coli* ATCC 25922 and *S. aureus* ATCC 29213, as the most resistant and sensitive bacteria respectively, were investigated (Avila et al., 1999). MHB was inoculated with test bacteria at 1% (v/v), and then treated with CEO and CEON at ½ MIC, followed by incubation at 37°C. The control contains DMSO 1% (v/v) instead of CEO and CEON. The optical density of microbial supernatants at 600_nm_ was measured by UV–VIS spectrophotometer for 9 h at 0.5 h intervals.

### 2.9 Mayonnaise preparation

The mayonnaise, as the control sample, was prepared according to Alizadeh et al. (2019) with modification containing the following ingredients (w/w): xanthan gum 0.4%, soybean oil 25%, carboxymethyl cellulose 0.4%, water 56.1%, egg yolk powder 0.5%, vinegar 9%, starch 1.5%, sugar 5%, salt 2%, and citric acid 0.1%. First, for aqueous phase preparation, the egg yolk powder, sugar, salt, and citric acid were homogenized with water for 4 min/10,000 rpm. Carboxymethyl cellulose, starch, and xanthan gum were mixed with oil and homogenized for 2 min/10,000 rpm. Then, this mixture and vinegar were slowly added to the aqueous phase and homogenized (8 min/1000 rpm) for emulsion formation. To prepare the treated samples, CEO (12 mg/mL) and CEON (6 mg/mL) were dissolved into the soybean oil and added to the mentioned recipe. The concentrations of CEO and CEON were chosen based on the results of antimicrobial (MBC) and antioxidant (DPPH radical‐scavenging assay) activities, then mayonnaises were distributed into small glass jars (200 gr). The jars, after sealing and labeling, were stored at ambient temperature (approximately 22°C) for 3 months. The mayonnaises were aseptically sampled at four‐time intervals (0, 1, 2, and 3 months) during storage for further analyses.

### 2.10 Determination of peroxide value

The peroxide value (PV) of mayonnaise samples was determined by iodometric titration according to Bligh and Dyer (1959) with slight modifications. For oil extraction from mayonnaise, 10 g of sample was mixed with 20 mL methanol and 10 mL chloroform for 2 min, then centrifuged at 2000 rpm for 10 min. The oil phase was separated, and residual solvents were removed using a rotary at 60°C. For peroxide assay, 1 g of potassium iodide was added to 1 g of extracted oil and then mixed with 20 mL solvent containing chloroform and acetic acid (2:3 ratio). After boiling the mixture for 30 s, 50 mL distilled water and 20 mL potassium iodide 5% were added, and then it was titrated by sodium sulfate (1/500 N) in the presence of starch solution. The peroxide value was measured as Equation (3).

(3) $$\mathrm{PV}\;\left(\mathrm{meq}\;O_{2}/\mathrm{kg}\;\text{oil}\right)=\frac{V\times N\times 1000}{M}$$

where V is the volume of expended sodium sulfate, N is the normality of sodium sulfate, and M is the sample weight.

### 2.11 Acid value (AV) assay

For AV determination, the oil dissolved in ethanol/chloroform was titrated with 0.1 N sodium hydroxide in the presence of phenolphthalein as an indicator, according to AOCS (1997). The AV was calculated as Equation (4).

(4) $$\mathrm{AV}\;\left(\%\right)=\frac{V\times N\times 28.2}{W}$$

where V is the volume of expended NaOH, N is the normality of NaOH, and W is the analyte weight.

### 2.12 Microbiological analysis

First, 5 g of each mayonnaise sample containing CEO, CEON, and control were mixed with 45 mL of sterile peptone water, and then serial dilutions up to 10^−7^ were prepared. The appropriate dilutions were plated on Orange Serum Agar (OSA, Merck, Germany) for *acid‐tolerant* organisms, on Plate Count Agar (PCA, Merck, Germany) for the total bacterial count, and on Potato Dextrose Agar (PDA, Merck, Germany) for fungi. PCA plates were incubated at 37°C for 48 h, OSA and PDA were incubated at 30°C and 25°C respectively, for 5 days (ISIRI, 2017; Pommerville, 2007).

### 2.13 Sensory analysis

The sensory properties of mayonnaise samples were evaluated at the end of 3 months of storage. The samples were assessed for color, taste, odor, texture, and overall acceptability based on the 5‐point hedonic scale (1 = least acceptable, 5 = most acceptable).

### 2.14 Statistical analysis

Statistical analysis was carried out by ANOVA (*p* < .05) using the SPSS, version 16. The significant differences between means were compared using the LSD test. All experiments were performed in triplicate.

## 3 RESULTS AND DISCUSSION

### 3.1 Chemical composition of CEO

The chemical compositions of the CEO are listed in Table 1. In the current study, Cuminaldehyde (27.99%), o‐Cymene (17.31%), γ‐Terpinen (16.67%), and β‐Pinene (9.35%) were the major components of the CEO from Northeastern Iran. Similarly, γ‐Terpinene, −β‐Pinene, and Cymene were identified as the dominant components of CEO from Northwestern Iran (Ghasemi et al., 2020). The chemical compounds of CEO can be attributed to the variations in cultivation method, climate conditions, and extraction process (Moosavi‐Nasab et al., 2016). The results of the current study were also consistent with those reported by Li and Jiang (2004), Akrami et al. (2015), Fasih et al. (2017), Nemati et al. (2019), Sharifi et al. (2021), and Ghannay et al. (2022).

**TABLE 1:** Chemical compositions of *Cuminum cyminum L*. essential oil.

Peak no.	Name	Molecular structure	Retention time	% of total
1	Cuminaldehyde	C_10_H_12_O	21.70	27.99
2	o‐Cymene	C_10_H_14_	12.48	17.31
3	Ƴ‐Terpinene	C_10_H_16_	14.20	16.67
4	β‐Pinene	C_10_H_16_	10.56	9.35
5	Silane, (4‐ethylpenyl) trimethyl‐	C_11_H_18_Si	27.56	7.22
6	3‐Caren‐ 10‐al	C_10_H_14_O	23.95	6.75
7	2‐Caren‐ 10‐al	C_10_H_14_0	23.61	5.78
8	1H‐3a,7‐Methanoazulene, 2,3,4,7,8,8a‐hexahydro‐3,6,8,8‐tetramethyl‐, [3R‐(3α,3aβ,7β,8aα)]‐	C_15_H_24_	32.36	1.83
9	Cuminic	C_10_H_12_O	20.53	0.99
10	Cyclopentene, 1,2,3,3‐tetramethyl‐4‐methylene‐	C_10_H_16_	29.34	0.97
11	Thymol	C_10_H_14_O	24.71	0.92
12	p‐Cymen‐7 –ol	C_10_H_14_O	24.16	0.86
13	Propanal, 2‐methyl‐3‐phenyl‐	C_10_H_12_O	20.80	0.85
14	Benzaldehyde, 4‐ (1‐methylethyl)‐	C_10_H_12_O	20.24	0.64
15	Eucalyptol	C_10_H_18_O	12.86	0.63
16	2‐Propanone, 1‐ (4‐methoxyphenyl)‐	C_10_H_12_O_2_	25.90	0.61
17	2‐Caren‐ 10‐al	C_10_H_14_O	23.46	0.61

### 3.2 Droplet size and microstructure of CEON

**FIGURE 1:**
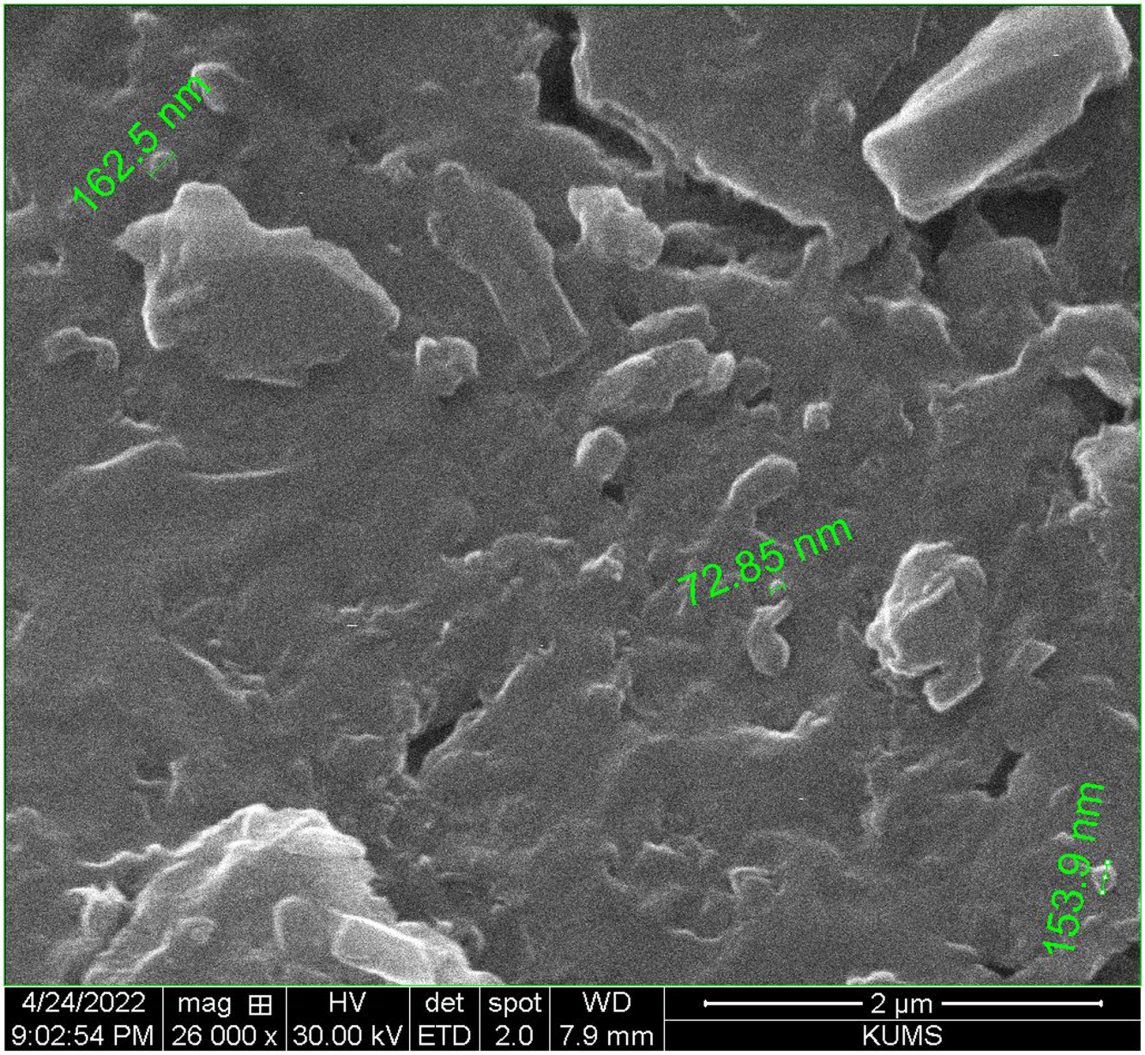
Scanning electron microscope (SEM) of *Cuminum cyminum L*. essential oil nanoemulsion prepared at 26,000× magnification.

In general, reducing the particle size leads to an increase in the surface area of particles (Csicsák et al., 2023), especially at the nanoscale. Reducing the average size of essential oil droplets in the nanoemulsion system increases the availability of bioactive compounds and as a result, the antioxidant and antimicrobial properties of the nanoemulsion compared with pure essential oil (Sharifi & Sharifi, 2023). Therefore, nanoemulsion particles with a size smaller than 100 nm are more suitable for greater utilization of their antimicrobial and antioxidant properties (McClements & Li, 2010). In this study, tween 80 was used as a surfactant for its high hydrophile–lipophile balance value to facilitate the formation of an oil‐in‐water emulsion. The small molecules of tween quickly absorb onto the CEO droplet surface and effectively reduce the diameter size of CEO droplets. In the current study, the average droplet diameter of the CEON was determined as 106.1 nm with a polydispersity index (PDI) of 0.445. Furthermore, the formation of CEON droplets at the nanoscale was confirmed using SEM (Figure 1). In similar studies, the mean droplet size of nanoemulsions was measured at 10.4, 70, and 155 nm for *Cuminum cyminum* essential oil (Nirmala et al., 2020), cumin essential oil (Rostami et al., 2018), and cumin seed oil (Farshi et al., 2017), respectively. Additionally, the droplet size of thyme essential oil nanoemulsion was reported to be 82.5–125.5 nm (Xue et al., 2015).

### 3.3 Antioxidant activity of CEO and CEON

The scavenging effects of CEO and CEON against DPPH radical were determined. According to our results, CEON exhibited stronger antioxidant activity with IC^50^ = 5 ± 0.07 (μg/mL) compared with CEO with IC^50^ = 10 ± 0.13 (μg/mL). The stronger antioxidant activity of the essential oil nanoemulsions can be related to the smaller size, greater solubility, and better permeability of droplets than essential oil emulsions; as a result, more free radicals are involved by the scavenging effects of essential oil nanoemulsions. Moreover, the pure essential oil is not able to dissolve in aqueous systems (Dhifi et al., 2016), which reduces its antioxidant activity compared with the nanoemulsion essential oil (Sharifi & Sharifi, 2023). However, nanoemulsions can dissolve in aqueous systems, which leads to the efficient release of activated compounds; subsequently, they can more effectively scavenge radicals (Lou et al., 2017). In previous studies, the IC^50^ (μg/mL) of *Cuminum cyminum L*. essential oil from different regions of Iran was reported as 23.3–32.4 (Karik et al., 2021) and 5.4 (Allahghadri et al., 2010). Differences in the antioxidant activity of essential oils can be attributed to phenolic compounds of plants; these chemical compositions are dependent on environmental conditions, plant genetics, and postharvest processes (Cowan, 1999; Vaya et al., 1997). According to previous studies, the antioxidant activity of the CEO under study can be attributed to its major components including Cuminaldehyde (Ghiasi et al., 2021), o‐Cymene (de Oliveira et al., 2015), Terpinen (Souza et al., 2018), β‐Pinene (Bouzenna et al., 2017; Salehi et al., 2019).

### 3.4 Antibacterial activity of CEO and CEON

The results of the antibacterial activity of CEO and CEON against *E. coli*, *P. aeruginosa*, *S. aureus*, and *B. cereus* are given in Table 2. The CEO and CEON exhibited considerable antibacterial activity against gram‐positive bacteria (*S. aureus* and *B. cereus*) compared with gram‐negative bacteria (*E. coli* and *P. aeruginosa*), so that *E. coli* (MIC_CEO_: 6, MIC_CEON_: 3; MBC_CEO_ 12, MBC_CEON_ 6 mg/mL) was the most resistant bacteria, while *S. aureus* (MIC_CEO_: 3, MIC_CEON_: 0.75; MBC_CEO_ 6, MBC_CEON_ 3 mg/mL) was the most sensitive bacteria. Most studies indicated that essential oils are slightly more effective against gram‐positive than gram‐negative bacteria (Burt, 2004; Chao et al., 2000; Mumivand et al., 2019). Gram‐negative bacteria are less sensitive to the inhibitory effect of essential oils due to an outer membrane in their cell wall (Ratledge & Wilkinson, 1988), which limits the distribution of hydrophobic compounds throughout the hydrophilic layer (Vaara, 1992). As cited in previous studies, the *Cuminum cyminum L*. Essential oil has antibacterial activity against different microorganisms, including *S. aureus* (Campana et al., 2022; Nirmala et al., 2020; Sharifi et al., 2021; Wongkattiya et al., 2019); *Vibrio* (Hajlaoui et al., 2010); *Candida albicans, Staphylococcus epidermidis, Bacillus subtilis, Saccharomyces cerevisiae* and *Aspergillus niger* (Jirovetz et al., 2005); *B. cereus* and *Salmonella typhi* (Wongkattiya et al., 2019). The high antimicrobial activity of CEO and CEON can be attributed to their major compounds, including Cuminaldehyde (Ghannay et al., 2022; Ghiasi et al., 2021; Wongkattiya et al., 2019), Terpinen (Bordini et al., 2018), and β‐Pinene (da Silva Rivas et al., 2012; Salehi et al., 2019). According to da Silva Rivas et al. (2012), pinenes, as one of the major components of CEO, can inhibit the activity of esterase and phospholipase of microorganisms. Furthermore, the results indicated that the inhibitory effect of nanoemulsified essential oil (CEON) was higher than that of pure essential oil (CEO). The sensitivity of test bacteria, especially *S. aureus*, to CEON is due to the fusion of the cellular lipid membranes with essential oils (Valgas et al., 2007). Zhang et al. (2009) reported that CEON leads to an increase in cytoplasmic leakage from pathogenic cells compared with the CEO. The fusion of CEON with the cellular lipid membrane leads to the degradation of cell membrane integrity; subsequently, the membrane permeability due to the destabilization of cellular structure causes increasing cytoplasmic leakage, and consequently cell death (Baker Jr et al., 2003).

**TABLE 2:** Minimum inhibitory concentrations (MIC) and *Minimum* Bactericidal Concentration (MBC) of CEO and CEON against food‐borne bacteria.

Microorganisms	MIC (mg/mL)		MBC (mg/mL)	
	CEO	CEON	CEO	CEON
*Pseudomonas aeruginosa* ATCC 9027	3	1.5	12	6
*Escherichia coli* ATCC 25922	6	3	12	6
*Staphylococcus aureus* ATCC 29213	3	0.75	6	3
*Bacillus cereus* ATCC 11778	3	1.5	6	3

Abbreviations: CEO, *Cuminum cyminum L*. essential oil; CEON, *Cuminum cyminum L*. essential oil nanoemulsion.

### 3.5 Kinetics assay of antibacterial activity

**FIGURE 2:**
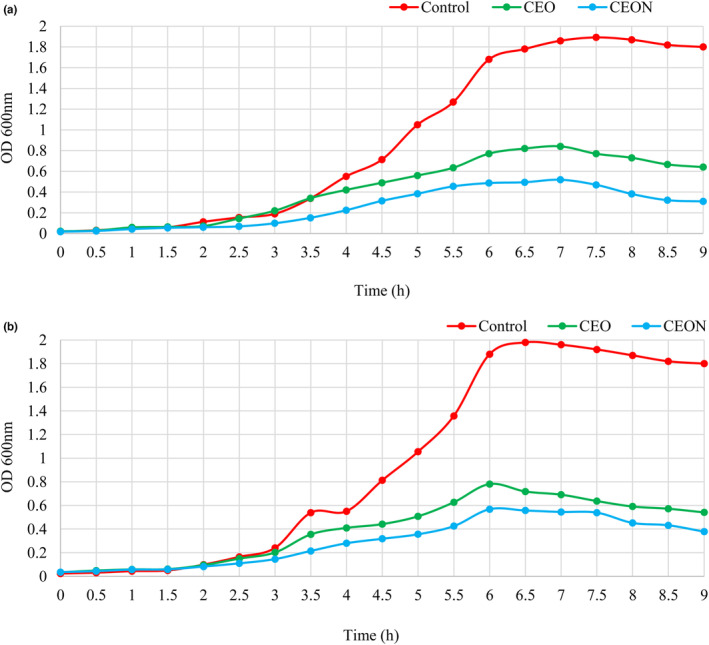
The growth curves of *E. coli* (a) and *S. aureus* (b) affected by CEO and CEON at ½ MIC in comparison with the control. CEO, *Cuminum cyminum L*. essential oil; CEON, *Cuminum cyminum L*. essential oil nanoemulsion; MIC, Minimum inhibitory concentration.

*Cuminum cyminum L*. essential oil nanoemulsions and CEO act as antibacterial agents when applied to *S. aureus* and *E. coli* at ½ MIC (Figure 2) in comparison with the control. The inhibitory effect of CEON and CEO does not exist during the first 7 h and 6 h for *E. coli* and *S. aureus*, respectively, but cell death slightly increases after this time; as a result, *E. coli* was more resistant than *S. aureus*. For both bacteria, the count of bacteria able to grow in the presence of CEON and CEO was considerably small in comparison with the control. Furthermore, the inhibitory effect of CEON on bacteria growth was more effective than CEO. The effective release of antimicrobial compounds from CEON and the large contact area of its droplets led to more reduction in the viable cell count than CEO during the incubation time.

### 3.6 Effect of CEON and CEO on peroxide value (PV)

**FIGURE 3:**
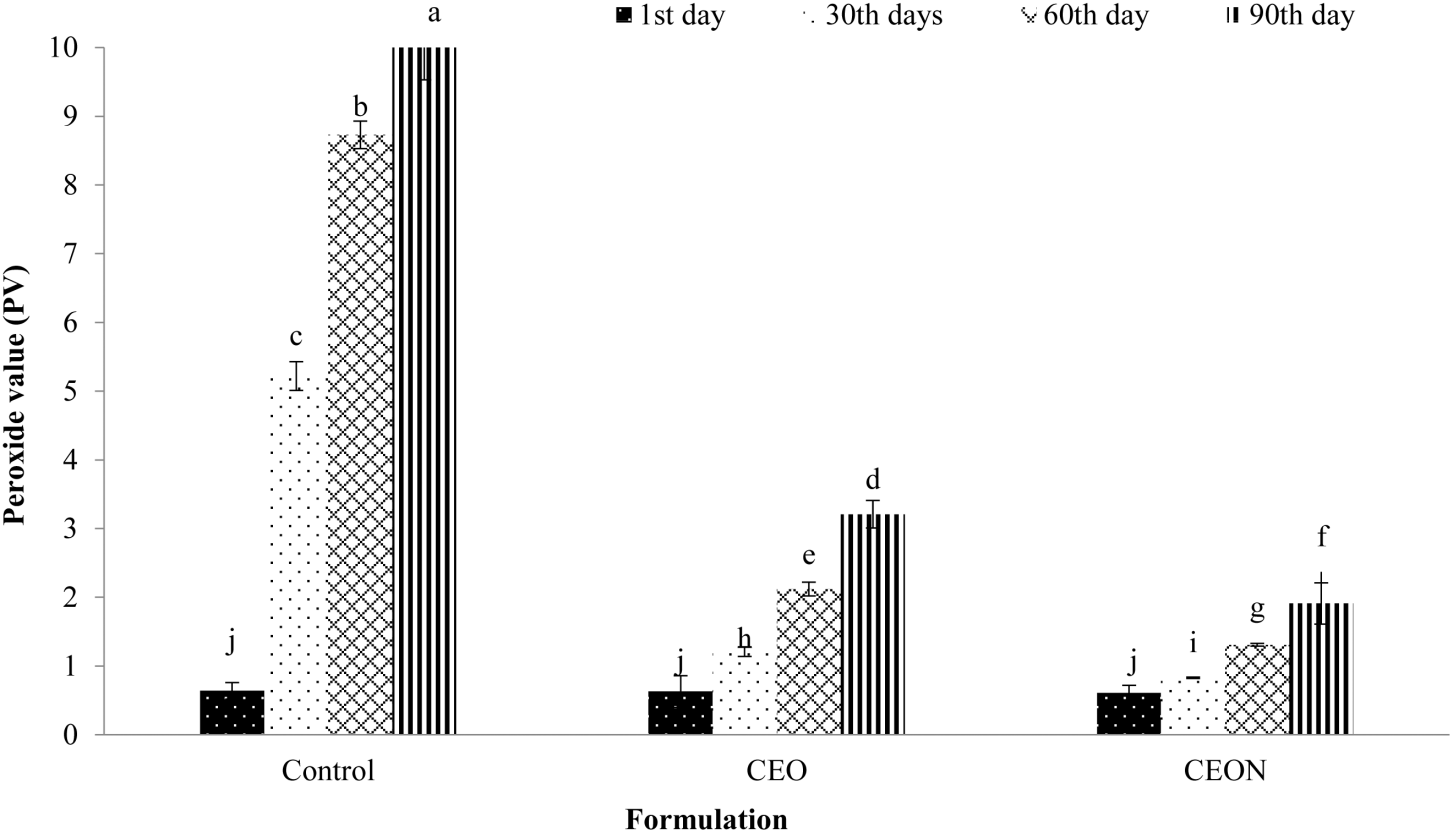
Effect of CEON and CEO on PV (meq O_2_/kg oil) of mayonnaise during 90 days of storage. Vertical bars represent the standard deviation (*n* = 3). Different letters indicate statistically significant differences (*p* < .05). CEO, *Cuminum cyminum L*. essential oil; CEON, *Cuminum cyminum L*. essential oil nanoemulsion.

The PV is measured to assess the primary oxidation of lipids by determining the concentration of hydroperoxides and peroxides. The results showed that the PVs of three treatments significantly (*p* < .05) increased during storage (Figure 3). In the third month, the PVs (meq O_2_/kg oil) in mayonnaises were CEON (1.91) < CEO (3.21) < Control (10.03); therefore, the PVs of CEON and CEO were lower in comparison with the control. The increase in PV is attributed to lipid oxidation in mayonnaise during storage (Sørensen et al., 2010). These findings were consistent with Gavahian et al. (2013), Kwon et al. (2015), and Alizadeh et al. (2019). Alizadeh et al. (2019) reported that the PV in mayonnaise containing tert‐Butylhydroquinone (TBHQ), as a strong commercial antioxidant, increased (7.74 meq O_2_/kg oil) during 3 months of storage. They also showed that the addition of rosemary essential oil significantly lowered the PV in mayonnaise compared with the control, although TBHQ was more effective. In comparison with Alizadeh et al. (2019), the results of the current study showed that *Cuminum cyminum L*. essential oil and its nanoemulsion have stronger antioxidant properties even than the commercial antioxidants such as TBHQ. This finding is in line with the results of Gavahian et al. (2013). They reported that the addition of Zenyan (Ajwain) essential oil to mayonnaise showed a considerable antioxidant effect in comparison with butylated hydroxyanisole (BHA) and butylated hydroxytoluene (BHT) (Gavahian et al., 2013). The antioxidant activity of essential oils can be due to the presence of phenolic compounds in these products, which react directly with free radicals resulting from the first stages of lipid oxidation (Gavahian et al., 2013; Guillén & Cabo, 2002). Kwon et al. (2015) indicated that tocopherol, as a natural antioxidant, could reduce PV from 7.84 to 2.30 meq O_2_/kg oil in mayonnaise during storage.

According to Kong and Singh (2011), PV should not exceed 10–20 (meq O_2_/kg oil) to avoid the rancid taste of food products containing oil. In the current study, PV increased in all samples during 3 months of storage. Therefore, the shelf life of mayonnaise containing CEO and CEON is not recommended to exceed 3 months.

### 3.7 Effect of CEON and CEO on acid value

**FIGURE 4:**
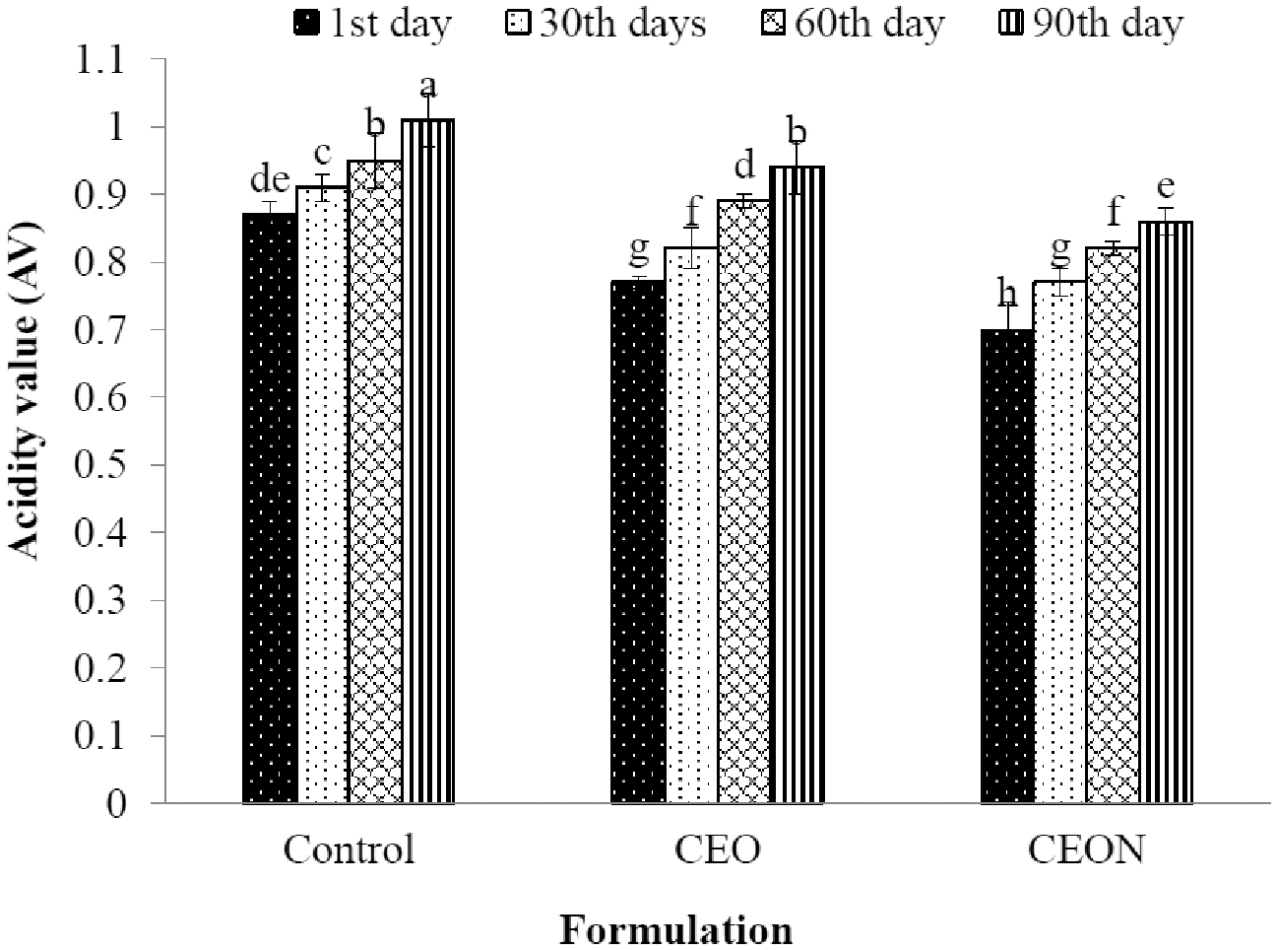
Effect of CEON and CEO on AV of mayonnaise during 90 days of storage. Vertical bars represent the standard deviation (*n* = 3). Different letters indicate statistically significant differences (*p* < .05). CEO, *Cuminum cyminum L*. essential oil; CEON, *Cuminum cyminum L*. essential oil nanoemulsion.

The acid value of oil extracted from mayonnaise is referred to the presence of carboxylic acid groups in fatty acids. The production of free fatty acids may be due to the breakdown of the ester groups of chemical compositions and then converting them into acidic compounds, oxidative reactions, hydrolysis of triglycerides, and microbial activity in the presence of water (Andres et al., 2005; Gavahian et al., 2013; Stephen & Phillips, 2016). As shown in Figure 4 the AVs of all samples significantly (*p* < .05) increased during 3 months of storage; however, the AVs in CEON and CEO were lower than the control. Similarly, the increasing trend of AV in mayonnaise was reported during the storage period according to Kishk and Elsheshetawy (2013) and Alizadeh et al. (2019). Alizadeh et al. (2019) indicated that among the mayonnaise samples containing natural and synthetic antioxidants (tertiary butylhydroquinone), adding rosemary essential oil led to the lowest AV.

The increase in AV can be related to the breakdown of iron connections with phosvitin in low pH during the storage period; consequently, released iron activates oxidative and hydrolytic reactions by enzymes in eggs (Honold et al., 2016; Kishk & Elsheshetawy, 2013). On the other hand, microbial activities in the nutrient matrix of mayonnaise, because of the nonthermal process, lead to organic acid production and lipid hydrolysis that subsequently increase AVs. Since the presence of essential oil in mayonnaise may inhibit microbial growth due to its antimicrobial properties and subsequently reduce the production of microbial organic acids, the AVs of CEO and CEON samples may be lower compared with the control sample. The findings of the current study proved this fact and indicated that the addition of CEO and CEON to mayonnaise affected the AV.

### 3.8 Effect of CEON and CEO on microbial growth

**FIGURE 5:**
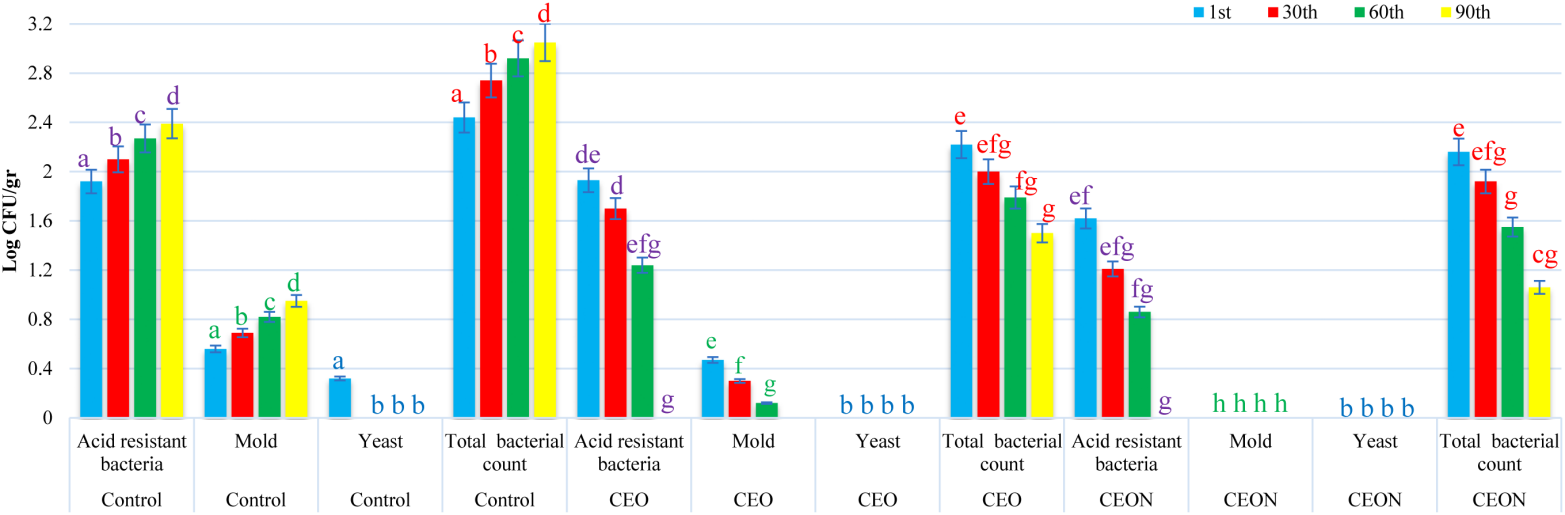
Effect of CEON and CEO on the microbial growth in mayonnaise samples during 90 days of storage. Vertical bars represent the standard deviation (*n* = 3). Different letters indicate statistically significant differences (*p* < .05); Purple letters: acid‐resistant bacteria, green letters: mold, blue letters: yeast, red letters: total bacterial count. CEO, *Cuminum cyminum L*. essential oil; CEON, *Cuminum cyminum L*. essential oil nanoemulsion.

The growth of microorganisms depends on the pH, temperature, and storage time of mayonnaise (Yolmeh et al., 2014). The essential oils can more effectively inhibit microbial growth when the pH of mayonnaise is low. This is because those easily dissolve in the lipid layer of the bacterial membrane in acidic mayonnaise due to their hydrophobic properties. As a result, the destruction of the bacterial membrane leads to the leakage of cellular compounds and the inhibition of cellular activities related to the membrane, resulting in cell death (Burt, 2004; da Silva & de Melo Franco, 2012; Holley & Patel, 2005; Lambert et al., 2001; Marchese et al., 2017; Smith‐Palmer et al., 2001). As shown in Figure 5, the growth of microorganisms changed in mayonnaise samples during the storage period. During 3 months of storage, the growth of acid‐resistant bacteria increased in the control sample and decreased in CEON and CEO samples; the growth of mold increased in the control sample and decreased in the CEO sample; the growth of yeast decreased in the control sample and was inhibited in the CEO sample, while the fungal growth was inhibited in the CEON sample; the total bacterial count increased in sample control and decreased in CEON and CEO samples. These findings are in line with the results of Smith‐Palmer et al. (2001), Iacobellis et al. (2005), Mizani and Gavami (2010), Ghorbani et al. (2015), El‐Kholany (2016), Marchese et al. (2017), Rezaloo et al. (2018), and Shahriari and Taghikhani (2018). As mentioned earlier, it was expected that CEON could more effectively inhibit the microorganism growth of mayonnaise than pure CEO during the storage period. It can be concluded from these results that the presence of *Cuminum cyminum* L. essential oil, especially at the nanoscale, effectively inhibits fungal growth and reduces the number of acid‐resistant bacteria and the total bacterial count compared with the control sample.

### 3.9 Sensory analysis of mayonnaise samples

**FIGURE 6:**
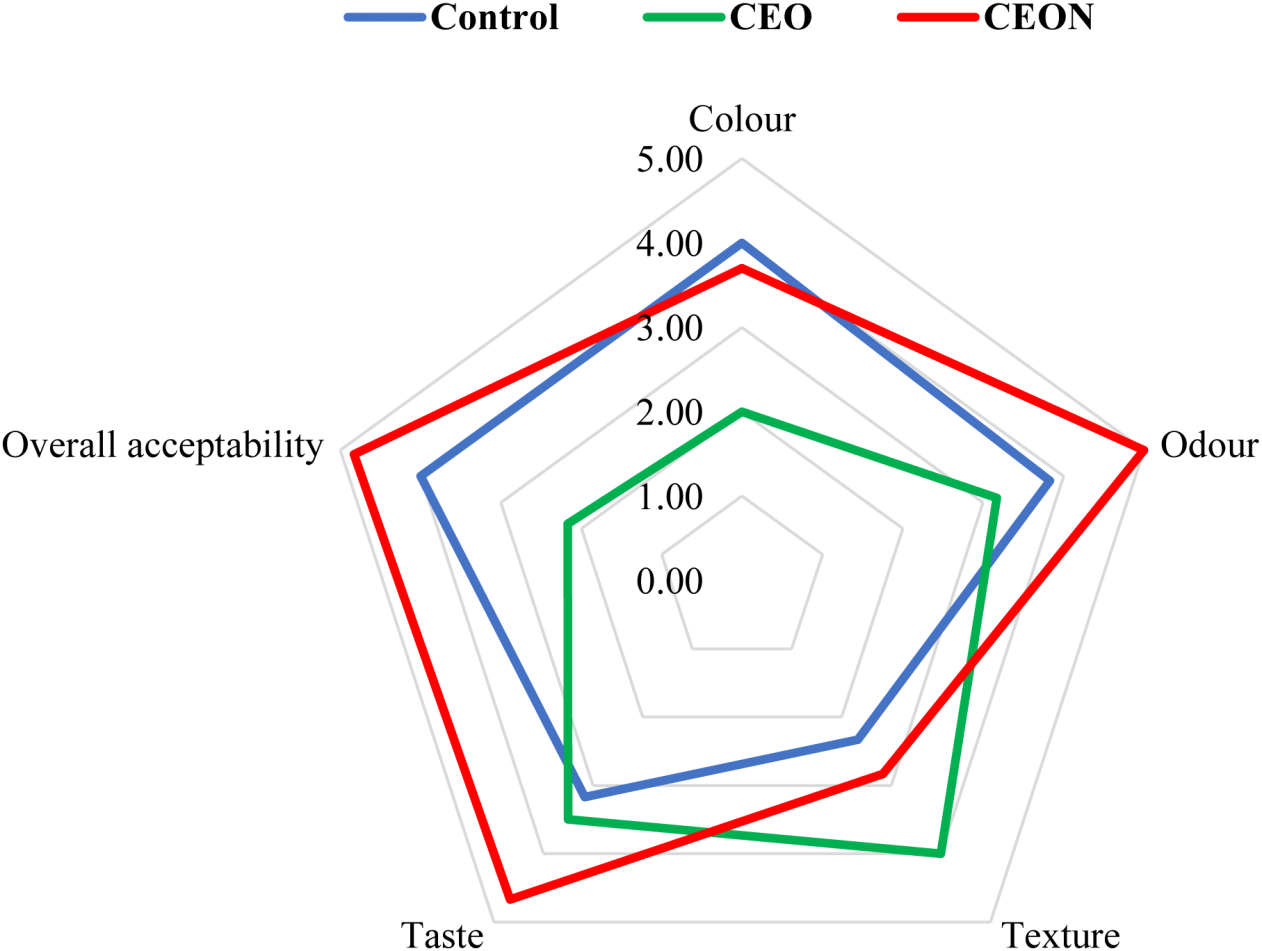
Consumer acceptability scores on a 5‐point scale for mayonnaise samples in the third month. CEO, *Cuminum cyminum L*. essential oil; CEON, *Cuminum cyminum L*. essential oil nanoemulsion.

Hydroperoxides, as colorless and tasteless volatile products, are responsible for the unpleasant smell and rancidity in food products containing oil (Alizadeh et al., 2019); therefore, the score of sensory attributes of these products is closely related to peroxide value during storage. During the storage period, the sensory attributes scores of the control sample significantly decreased; while the odor and taste scores of CEON and CEO samples significantly increased according to Data S1. The decreasing trend of odor and taste scores for the control sample can be associated with the progression of oxidation throughout 3 months of storage; however, these oxidative reactions have been inhibited due to the antioxidant activity of essential oils for CEON and CEO samples. As shown in Figure 6 the color of mayonnaise samples was affected by essential oil concentration, so this score was decreased in the CEO sample compared with the CEON and control samples in the third month. The highest score for odor (5), taste (4.67), and overall acceptability (4.83) was obtained for the CEON sample, and the lowest score for color (2.00), odor (3.17), and overall acceptability (2.17) was determined for CEO sample after 3 months of storage. The lower scores of sensory attributes of the CEO sample could be attributed to the high concentration of essential oil (12 mg/mL) in comparison with CEON (6 mg/mL) and control samples, which leads to an undesirable color and unpleasant odor. Similarly, Mansouri et al. (2021) reported that adding *Thymus daenensis L*. essential oil nanoemulsion to mayonnaise led to an increase in sensory scores in comparison with pure essential oil.

It can be concluded from these results that the low concentration of *Cuminum cyminum L*. essential oil at the nanoscale effectively improves the odor, taste, and overall acceptability of mayonnaise in comparison with the sample containing the high concentration of *Cuminum cyminum* L. essential oil and control sample.

## 4 CONCLUSION

In this study, the efficacy of *Cuminum cyminum L*. essential oil and its nanoemulsion on the microbial growth and oxidation stability of mayonnaise was investigated during 3 months of storage. The antioxidant and antimicrobial activities of CEON and CEO were confirmed. The addition of CEON and CEO in mayonnaise inhibited microbial growth and improved the oxidation stability (PV) in comparison with the control sample during the storage period. Although, CEON was more efficient than CEO in controlling the microbial growth and oxidative changes due to more efficiency and better performance of emulsion droplets of essential oil at the nanoscale. The evaluation of sensory attributes of mayonnaises indicated that the CEO sample obtained lower scores than CEON and control samples due to its undesirable color and odor. Based on the results, *Cuminum cyminum L*. essential oil nanoemulsion, due to its desirable sensorial characteristics, oxidative stability, and controlling microbial growth, can be recommended as an appropriate alternative to synthetic antioxidants and preservatives in food products.

## AUTHOR CONTRIBUTIONS

**Asma Moradi:** Conceptualization (equal); data curation (equal); formal analysis (equal); investigation (equal); methodology (equal); validation (equal). **Nafiseh Davati:** Conceptualization (lead); data curation (lead); formal analysis (lead); funding acquisition (supporting); investigation (lead); methodology (lead); project administration (lead); resources (supporting); software (supporting); supervision (equal); validation (lead); visualization (equal); writing – original draft (equal); writing – review and editing (equal). **Aryou Emamifar:** Methodology (supporting); project administration (lead); supervision (lead).

## FUNDING INFORMATION

This research did not receive any specific grant from funding agencies in the public, commercial, or not‐for‐profit sectors.

## CONFLICT OF INTEREST STATEMENT

The authors declare that they do not have any conflict of interest.

## ETHICAL APPROVAL

This study does not involve any human or animal testing.

## CONSENT TO PARTICIPATE

All the coauthors were willing to participate in this manuscript.

## CONSENT FOR PUBLICATION

All authors are willing for the publication of this manuscript.

## Supporting information


Data S1.

## Data Availability

Even though adequate data have been given in the form of tables and figures, all authors declare that if more data are required, then the data will be provided on a request basis.
